# Use of biomaterials for sustained delivery of anti-VEGF to treat retinal diseases

**DOI:** 10.1038/s41433-020-0770-y

**Published:** 2020-01-30

**Authors:** Ivan Seah, Xinxin Zhao, Qianyu Lin, Zengping Liu, Steven Zheng Zhe Su, Yew Sen Yuen, Walter Hunziker, Gopal Lingam, Xian Jun Loh, Xinyi Su

**Affiliations:** 1 Department of Ophthalmology, National University Hospital Singapore, Singapore, Singapore; 2 Institute of Molecular and Cell Biology (IMCB), Agency for Science, Technology and Research (A*STAR), Singapore, Singapore; 3 Institute of Materials Research and Engineering (IMRE), Agency for Science, Technology and Research (A*STAR), Singapore, Singapore; 4 Department of Ophthalmology, Yong Loo Lin School of Medicine, National University Singapore, Singapore, Singapore; 5 Singapore Eye Research Institute, Singapore, Singapore; 6 Lee Kong Chian School of Medicine, Nanyang Technological University, Singapore, Singapore; 7 Department of Physiology, National University of Singapore, Singapore, Singapore

**Keywords:** Implants, Macular degeneration, Retinal diseases, Antibody therapy, Drug therapy

## Abstract

Anti-vascular endothelial growth factors (anti-VEGF) have become the most common treatment modality for many retinal diseases. These include neovascular age-related macular degeneration (n-AMD), proliferative diabetic retinopathy (PDR) and retinal vein occlusions (RVO). However, these drugs are administered via intravitreal injections that are associated with sight-threatening complications. The most feared of these complications is endophthalmitis, a severe infection of the eye with extremely poor visual outcomes. Patients with retinal diseases typically have to undergo multiple injections before achieving the desired therapeutic effect. Each injection incurs the risk of the sight-threatening complications. As such, there has been great interest in developing sustained delivery platforms for anti-VEGF agents to the posterior segment of the eye. In recent years, there have been various strategies that have been conceptualised. These include non-biodegradable implants, nano-formulations and hydrogels. In this review, the barriers of drug delivery to the posterior segment of the eye will be explained. The characteristics of an ideal sustained delivery platform will then be discussed. Finally, the current available strategies will be analysed with the above-mentioned characteristics in mind to determine the advantages and disadvantages of each sustained drug delivery modality. Through the above, this review attempts to provide an overview of the sustained delivery platforms in their various phases of development.

## 摘要

抗血管内皮生长因子 (Anti-vascular endothelial growth factors, anti-VEGF) 已成为治疗视网膜疾病最常见的治疗方法, 其中包括新生血管性年龄相关性黄斑变性 (neovascular age-related macular degeneration, n-AMD) 、增殖型糖尿病视网膜病 (proliferative diabetic retinopathy, PDR) 和视网膜静脉阻塞 (retinal vein occlusions, RVO) 。然而, 给药途径为玻璃体腔内注射, 因此可能会发生威胁视力的并发症。其中最可怕的并发症是眼内炎。眼内炎为严重的眼部感染, 视力预后极差。罹患视网膜疾病的患者通常需要多次眼内注射达到预期的治疗效果。每次注射都会有威胁视力并发症发生的风险。因此, 人们对抗VEGF药物应用于眼后节的持续性给药系统的研发非常关注。近年来, 新的治疗策略新的治疗理念不断涌现, 其中包括非生物降解性植入物、纳米制剂和水凝胶。本文我们将阐述药物输送至后节的屏障, 随后探讨理想的持续性输送给药系统的特点, 最后将结合上述特点分析现有的策略, 以确定每种持续性药物输送方式的优缺点。本文旨在综述持续性输送给药系统的各个发展阶段的概况。

## Introduction

Anti-vascular endothelial growth factors (anti-VEGF) have become the most common treatment modality for many retinal diseases. These include proliferative vascular disorders such as neovascular age-related macular degeneration (n-AMD), proliferative diabetic retinopathy (PDR) and retinal vein occlusions (RVO). Anti-VEGF is also used to treat retinal conditions involving the disruption of the blood–retinal barrier such as diabetic macular oedema (DMO). If not treated, these conditions can cause profound visual impairment, resulting in a significant reduction in quality of life. To deliver the drug directly to the retina, anti-VEGF agents are administered intravitreally. This reduces the dosage required and avoids the systemic side effects associated with systemic administration [1].

These intravitreal injections are now the most commonly performed ophthalmic procedures [2]. However, intravitreal injections are associated with various complications. The most feared complication is the development of endophthalmitis, a sight-threatening infection with reported incidences of between 0.019 and 0.09% [3, 4]. Unfortunately, in many of the retinal diseases mentioned, multiple monthly to bi-monthly injections are required for long periods of time, thus increasing the risk of infection.

Apart from the endophthalmitis risk, the need for frequent injections also poses a significant burden to the patient and their caregivers [5, 6], with some patients needing up to a day of recovery after their injections, resulting in the loss of work productivity [5].

There has been great interest in developing sustained anti-VEGF delivery systems in a bid to reduce the frequency and risks of multiple intravitreal injections. This review highlights the delivery-related limitations of anti-VEGF compounds and discusses the innovative research strategies to overcome these issues. The review will have a particular focus on novel hydrogel technologies as they have shown great potential as sustained drug delivery platforms.

## VEGF-dependent pathophysiology of common retinal vascular diseases

With an ageing population, retina vascular diseases are becoming more commonly diagnosed. These conditions are associated with either pathological retinal neovascularisation or oedema from the disruption of the blood–retinal barrier. Among these conditions, n-AMD, PDR, DMO and RVO represent the leading causes of visual impairment globally [7, 8]. VEGF has been shown to play an important role in the pathophysiology of these diseases. In both DMO and PDR, intravitreal anti-VEGF is used to target the VEGF-driven processes of neovascularisation [9] and blood–retinal-barrier dysfunction [10]. The treatment has been used extensively with proven benefits [11]. In RVO, anti-VEGF agents have also been effective at treating cystoid macular oedema [12–16], a condition that occurs due to VEGF-driven BRB breakdown [17]. Finally, all three of the currently available anti-VEGF drugs Bevacizumab (Avastin™), Ranibizumab (Lucentis™) and Aflibercept (Eylea™) are also proven first-line therapies for n-AMD and are extensively used worldwide for the treatment of the VEGF-driven complication [18] of choroidal neovascularisation (CNV) [19, 20].

## Anti-VEGF agents for retinal vascular diseases

Anti-VEGF drugs were first discovered in the 1970s to combat the mechanism of tumour angiogenesis, a key process contributing to tumour proliferation and metastasis. In 2004, the first anti-VEGF agent, Bevacizumab (Avastin™) was approved for the combination treatment with chemotherapy for colon cancer. Along with that development, the first anti-VEGF therapy for retinal vascular conditions was developed. This was pegaptanib sodium (Macugen™), an RNA aptamer that binds and neutralises selectively VEGF-165 in the treatment of n-AMD [21]. Shortly thereafter, systemic administration of Bevacizumab (Avastin™) was also found to be effective in the treatment of n-AMD [1]. This prompted many ophthalmologists to begin injecting the drug intravitreally for better results [22].

Anti-VEGF treatments have revolutionised the treatment of the above-mentioned diseases. Since the initial usage of Bevacizumab (Avastin™), efforts into developing new anti-VEGF agents continued. Currently, three drugs available Bevacizumab (Avastin™), Ranibizumab (Lucentis™) and Aflibercept (Eylea™). Table 1 highlights the currently available anti-VEGF treatments and their main characteristics.

**Table 1:** Available anti-VEGF compounds and their properties [28].

Drug	Type of molecule	Molecular weight	Half-life in posterior segment of eye	Typical drug dosage per injection
Bevacizumab (Avastin™)	Recombinant humanised IgG1 monoclonal antibody inhibitor of VEGF-A	149 kDa	~8 days	1.25 mg in 0.05 ml
Ranibizumab (Lucentis™)	Recombinant humanised IgG1 monoclonal antibody fragment inhibitor of VEGF-A	48 kDa	~5 days	0.5 mg in 0.05 ml
Aflibercept (Eylea™)	Decoy receptor inhibitor of VEGF-A and VEGF-B	115 KDa	~7 days	2.0 mg in 0.05 ml

## Limitations of anti-VEGF agents in the treatment of retinal vascular diseases

While effective at controlling neovascularisation, vascular leakage and other pathological changes associated with retinal vascular diseases, anti-VEGF agents also have their limitations. These limitations stem from the necessity for multiple intravitreal administrations in order to achieve their optimal and continued effect. As opposed to topical administration via eye drops and ointments, intravitreal administration can potentially lead to ocular complications ranging from subconjunctival haemorrhage, raised intraocular pressure to sight-threatening endophthalmitis and rhegmatogenous retinal detachment [23].

**Fig. 1:**
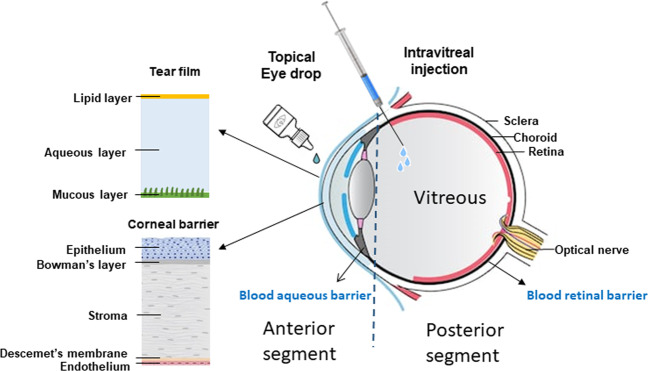
Barriers to anti-VEGF delivery to the retina. When delivered topically, anti-VEGF compounds will have to overcome various barriers. This includes physical barriers such as the five layers of the cornea, the blood aqueous barrier and the vitreous before it reaches the retina. Dynamic barriers include the tear film and systemic clearance from conjunctival blood vessels.

To date, alternative methods of administration have been unsuccessful. In contrast to intravitreal administration, topical delivery of adequate concentrations of anti-VEGF agents to the retina remains challenging due to multiple barriers against drug penetration. When administered topically, anti-VEGF agents have to traverse the various layers of the cornea, bypass multiple dynamic barriers and penetrate the inner layers of the retina in order to reach the blood vessels. Dynamic barriers include dilution of medication by the tear film and systemic clearance through conjunctival blood vessels. Structural barriers include the five different layers of the cornea. In the cornea epithelium, tight junctions and lipophilicity prevent hydrophilic molecules from penetrating through [24]. Even if the molecule reaches the stromal layer, collagen fibres that are highly organised and intertwined with narrow pore size can prevent further permeation. These barriers result in less than 5% of topical medications reaching the anterior segment of the eye [25]. Once in the anterior segment, drug transporters in the iris–ciliary body also actively eliminate drug from the aqueous humour, reducing its ocular bioavailability [26]. Figure 1 illustrates the various barriers of delivery associated with topical administration of anti-VEGF compounds.

Other methods of drug administration have also been proposed, including trans-conjunctival or trans-scleral routes [27]. However, their utility in clinical practice for anti-VEGF administration is limited. Drugs administered in this manner diffuse via the conjunctiva through the sclera into the choroid before reaching the retina. Along this path, the choroidal circulation and BRB result in rapid drug clearance, leading to sub-therapeutic dosages reaching the retina.

In view of the inherent difficulties in administering anti-VEGF compounds via alternative routes, intravitreal administration still remains the only effective method. However, anti-VEGF compounds have short half-lives in the posterior segment of the eye, necessitating the need for multiple injections. The half-life of current available anti-VEGF compounds range from ~5–8 days (Table 1) [28]. This need for frequent injections not only exposes patients to the risk of endophthalmitis but also results in high treatment dropout rates. Since the advent of intravitreal anti-VEGF therapy, there has also been a significant increase in clinic visits and subsequent economic burden to healthcare systems worldwide [29]. The provision of intravitreal anti-VEGF therapy created a rapid increase in healthcare workload, which some healthcare systems may find difficult to cope with. This, in addition to the issue of patient compliance, results in a problem of inadequate treatment. It has been shown that patients generally received less injections than that given in clinical trials for n-AMD [30, 31], DMO and RVOs [31–33]. This limits the effectiveness of anti-VEGF therapy in retinal vascular diseases [30–36].

Various strategies to reduce treatment burden have been investigated. These include photodynamic therapy, which has been tried as a combination therapy with anti-VEGF agents, but this has been found to be of limited use in the SUMMIT, DENALI and MONT BLANC trials [37–39]. Similarly, radiation therapy has been tried as an addendum to anti-VEGF drugs, but this has also failed to reach the stated objective in clinical trial (CABERNET trial) [40].

Treat-and-extend regimes have been explored as a dosing strategy to reduce total number of injections required [41]. In this regime, patients receive regular intravitreal anti-VEGF injections with progressively longer intervals between treatments as long as the macula remains dry and stable. To date, the longest interval achieved with the treat-and-extend regime with an anti-VEGF drug was in the ALTAIR trial [42], where 40% of patients achieved a 16 week interval between their injections of Aflibercept for ARMD.

There has also been great interest in new anti-VEGF compounds with longer half-lives, which would enable longer intervals between treatments. The latest anti-VEGF agent, Brolucizumab, a humanized single-chain antibody fragment for the treatment of n-AMD, is currently undergoing US Food and Drug Administration (FDA) review for potential release by the end of 2019. Due to regulatory restrictions, Brolucizumab has only been investigated for a maximum interval of 12 weeks after an initial loading dose of three monthly injections in the HAWK and HARRIER trials [43, 44]. While future trials with longer intervals may reveal an even longer half-life, with the current available evidence, a solution that can reduce the frequency of intravitreal injections may remain elusive.

As such, there is a clear unmet clinical need for the development of sustained delivery methods for anti-VEGF molecules in the treatment of retinal vascular diseases. This will not only improve the safety profile of anti-VEGF treatments, but also reduce the burden of treatment on the patient, caregiver and healthcare system.

## Novel sustained drug delivery systems for anti-VEGF agents

**Fig. 2:**
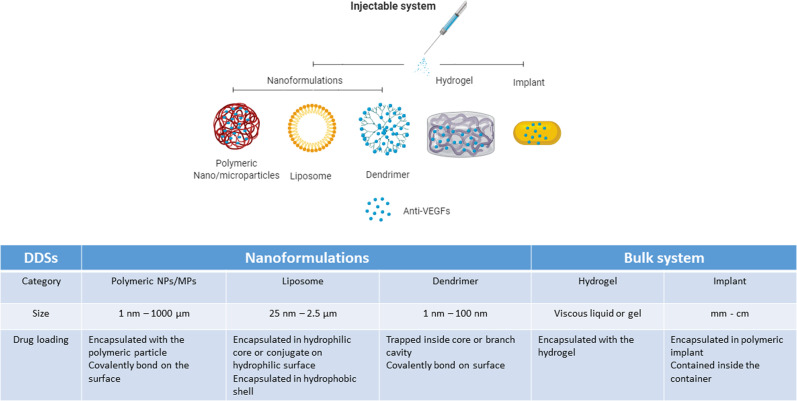
Promising strategies for sustained anti-VEGF delivery to the retina.

To date, non-biodegradable implants, nano-formulations and hydrogels have emerged as promising strategies (Fig. 2) [45]. Hydrogels have been used as promising materials for a variety of ophthalmic applications [46–53]. However, developing sustained drug delivery systems for biologics such as anti-VEGF molecules is a challenging task. Anti-VEGF molecules are proteins that require the preservation of their fragile tertiary and quaternary structures for activity. Hence, they are sensitive to various environmental factors, including heat, pH changes and proteolytic enzymes [54]. These molecules are also membrane impermeable, making delivery of the drug a challenge.

**Fig. 3:**
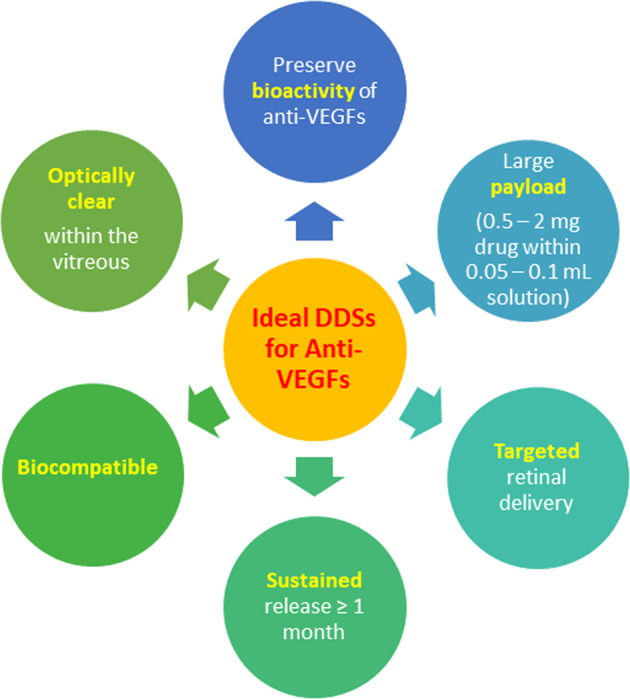
Ideal characteristics of a sustained anti-VEGF delivery platform.

An ideal biomaterial for sustained anti-VEGF delivery to the retina will need to fulfil certain characteristics (Fig. 3). Firstly, the biomaterial has to be able to preserve the bioactivity of the anti-VEGF molecule by protecting the tertiary and quaternary structure of the protein from denaturation, pH changes or enzymatic degradation. It also has to be able to deliver the anti-VEGF molecule to the retina. To avoid intraocular pressure elevation on administration, the biomaterial needs to be able to encapsulate a large amount of anti-VEGF in a minimal volume (~0.5–2 mg anti-VEGF within a maximum volume of 0.05–0.1 ml). After administration, the biomaterial should sustain the release of anti-VEGF for more than 1 month to avoid multiple administrations. The duration of 1 month has been proposed as the half-life of anti-VEGF agents currently administered intravitreally range between 5 and 8 days (Table 1). Lastly, the delivery system should be biocompatible and remain optically clear within the vitreous to avoid any interference with vision.

Given the necessity of the above characteristics, it is no surprise that the authors of this article were not able to identify any US-FDA approved sustained anti-VEGF delivery systems to the posterior segment of the eye when a search of the 510k and pre-market approval databases was conducted. This review will discuss the sustained anti-VEGF delivery strategies that the authors are aware of and that are currently in development, as well as their ability to meet the above requirements (Table 2).

**Table 2:** Novel sustained anti-VEGF [Bevacizumab (Avastin™), Ranibizumab (Lucentis™) and Aflibercept (Eylea™)] delivery systems tested in in vivo models.

Technology and year of publication	Drug loaded	Phase of development	Bioactivity testing	Biocompatibility testing	Sustained release testing and duration	Drug encapsulation characteristics (encapsulation efficiency and loading efficiency)	Optical clarity
Polymeric nanoparticle (NP) and microparticle (MP) technology							
Drug-loaded PLGA/PCADK microspheres (2019) [60]	Bevacizumab (Avastin™)	Pre-clinical • SR tested In vivo • Biocomp tested in vivo	In vitro bioactivity proven on chorioallantoic membrane assay	New Zealand white rabbits • Mild inflammatory cells on histology	New Zealand white rabbits IVT • At least 50 days	EE: 35% for 20% PCADK content	Not commented
Drug-loaded mesoporous silica nanoparticles (2019) [62]	Bevacizumab (Avastin™)	Pre-clinical • SR tested in vivo • Biocomp tested in vivo • Bioactivity tested in vivo	Proven on oxygen-induced retinopathy mouse model	C57BL/6J mice • NIL histological changes • NIL significant difference in ERG findings with control	C57BL/6J mice IVT • Half-life of 8.7 days as opposed to 5.3 days in direct injections	EE: 85.3%	Not commented
Drug-loaded PLGA microspheres (2015) [63]	Bevacizumab (Avastin™)	Pre-clinical • SR tested in vivo	NIL bioactivity testing	NIL biocompatibility results	New Zealand albino rabbit IVT • At least 42 days	EE: 49% LE: 9.8%	Not commented
Drug-loaded albuminated PLGA nanoparticles (2015) [61]	Bevacizumab (Avastin™)	Pre-clinical • SR tested in vivo • Biocomp tested in vivo	NIL bioactivity testing	New Zealand albino rabbits • NIL inflammation on histology • NIL changes to ERG	New Zealand albino rabbit IVT • At least 2 months	EE: 84% LE: 7.4%	Not commented
Drug-linked chitosan nanoparticles (2014) [58]	Bevacizumab (Avastin™)	Pre-clinical • SR tested in vivo • Biocomp tested in vivo	RT PCR assay testing of VEGF mRNA expression in diabetic rat retina	NIL biocompatibility results	Sprague Dawley diabetic rats • At least 8 weeks	Not commented	No commented
Drug-loaded PLA nanoparticles in porous PLGA microparticles (2013) [64]	Bevacizumab (Avastin™)	Pre-clinical • SR tested in vivo	NIL bioactivity testing	NIL biocompatibility results	Rat model IVT • At least 4 months	NIL LE or EE data	Not commented
Liposome technology							
Drug-loaded multi-vesicular liposomes (2018) [65]	Bevacizumab (Avastin™)	Pre-clinical • SR tested in vivo • Bioactivity tested in vivo	Proven on laser-induced choroidal neovascularisation Brown-Norway rat model	NIL biocompatibility results	New Zealand white rabbit IVT • At least 56 days	EE: 80.6% • LE: not stated	Not commented
Drug-loaded egg phosphatidylcholine:cholesterol (liposome) (2009) [68]	Bevacizumab (Avastin™)	Pre-clinical • SR tested in vivo	NIL bioactivity testing	NIL biocompatibility results	New Zealand albino rabbit IVT • At least 42 days (concentration of drug still five times higher than Bevacizumab solution)	EE: 45.5% LE: not stated	Not commented
Hydrogel technology							
Drug-loaded PLGA microspheres suspended in a PEG-PLLA-DA/NIPAAm hydrogel (2019) [99, 103, 104]	Ranibizumab (Lucentis™)Aflibercept (Eylea™)	Pre-clinical • SR tested in vivo • Biocomp tested in vivo • Bioactivity tested in vivo	Proven on laser-induced choroidal neovascularisation Long-Evans rat model	Long-Evans Rat model • Small transient increase in intraocular pressure after injection • NIL changes on ERG	Long-Evans rat IVT • At least 12 weeks	Ranibizumab microsphere: • EE in microsphere: 45.6% • EE of microsphere in gel: 74.2% • Aflibercept microsphere • EE in microsphere: 52% • EE of microsphere in gel: 70.9% Aflibercept	Not commented
OTX-IVT (anti-VEGF intravitreal hydrogel implant) (2017) [108]	Bevacizumab (Avastin™)	Pre-clinical • SR tested in vivo • Bioactivity tested in vivo • Biocomp tested in vivo • Clinical trials have commenced for tyrosine kinase inhibitor (TKI) loaded-gel (ClinicalTrial.gov ID: NCT03630315)	Proven on rabbit VEGF challenge model	Rabbit VEGF challenge model • NIL evidence of inflammation	Rabbit IVT VEGF challenge model • At least 12 weeks	Not commented	Compact depot which does not cause blurring of vision
Drug-loaded PLGA-PEG-PLGA hydrogel (2015) [100]	Bevacizumab (Avastin™)	Pre-clinical • SR tested in vivo • Biocomp tested in vivo	NIL neovascularisation models tested	Sprague Dawley rats • NIL signs of inflammation clinically and histologically • NIL changes to retinal thickness on OCT • NIL changes on ERG at 4 or 8 weeks	Sprague Dawley rats IVT • At least 6 weeks	1.25% drug content gel created	Not commented
Drug-loaded vinylsulfone functionalised hyaluronic acid (HV-VS) and thiolated dextran (Dex-SH) hydrogel (2015) [92]	Bevacizumab (Avastin™)	Pre-clinical • SR tested in vivo • Biocomp tested in vivo	In vitro bioactivity proven on ELISA assay with VEGF-capture protein	New Zealand white rabbits • Transient increase in intraocular pressure after injection. • NIL observed abnormalities on BIO • NIL changes on ERG • NIL signs of inflammation histologically	New Zealand white rabbits IVT • At least 6 months (drug concentration 10^7^ times higher than eyes receiving bolus injections)	12.5 mg/ml drug content gel created • 40 µl gel injected	Transparent as viewed on the BIO
Drug-loaded silk hydrogel (2015) [91]	Bevacizumab (Avastin™)	Pre-clinical • SR tested in vivo • Biocomp tested in vivo	In vitro bioactivity proven on ELISA assay with VEGF-capture protein	Dutch-belted rabbits • Minimal inflammation noted clinically in AC/posterior segment after 1 month	Dutch-belted rabbits IVT • At least 3 months	Standard dose hydrogel • 1.25 mg in 50 µl gel High-dose hydrogel • 5 mg in 50 µl gel	Not commented
Drug-loaded poly (ethylene glycol)-poly-(serinol hexamethylene urethane) (ESHU) hydrogel (2014) [98, 101]	Bevacizumab (Avastin™)	Pre-clinical • SR tested in vivo • Biocomp tested in vivo	NIL neovascularisation models tested	New Zealand white rabbit • NIL evidence of inflammation clinically or on histology • NIL change in IOP post injection	New Zealand white rabbits IVT • At least 9 weeks (drug conc 4.7 times higher than eyes receiving bolus injections)	Not commented	Not commented
Non-biodegradable implant technology							
Port delivery system (PDS) (2016) [56]	Ranibizumab (Lucentis™)	Clinical • Phase III trials (Pivotal)	Ranibizumab-responsive n-AMD patients • 100 mg/ml PDS produces similar visual acuity as monthly injections	Ranibizumab-responsive n-AMD patients • PDS arm had more adverse events than monthly injection group	Average refill time • Every 16 months	Not applicable	Not applicable
Replenish Posterior Micropump (PMP) (2014) [55]	Ranibizumab (Lucentis™)	Clinical • Phase I trials (Feasibility)	Diabetic macular oedema patients • Decrease in central foveal thickness at 2nd week	Diabetic macular oedema patients • NIL serious adverse events or visual acuity loss noted	Trial lasted for 3-month period	Not applicable	Not applicable

Encapsulation efficiency (EE%) = (Total drug added−free non-entrapped drug)/total drug added. Loading efficiency (LE%) = Amount of total entrapped drug/total nanoparticle weight.

*SR* sustained release, *OTX* ocular therapeutix, *Biocomp* biocompatibility, *IVT* intravitreal injection, *IOP* intraocular pressure, *EE* encapsulation efficiency, *LE* loading efficiency, *PLLA* poly (l-lactic acid), *NIPAAm* N-isopropylacrylamide.

## Non-biodegradable implants

Among the various platforms of sustained anti-VEGF delivery systems for the posterior segment of the eye, non-biodegradable implants are closest to achieving clinical translation. These implants are devised mainly as intravitreal depots containing anti-VEGF medication, allowing for a prolonged diffusion of anti-VEGF medication to the posterior segment. These delivery systems usually require a surgical implantation procedure. They can then be refilled in the clinic setting when the depot runs out of the drug of choice.

The Posterior Micropump (PMP) drug delivery system by Replenish, Inc^®^ is a programmable micropump designed to hold 0.6 ml of an anti-VEGF agent. The device is implanted under the conjunctiva and tenons. The anti-VEGF agent is actively pumped by a microelectromechanical system (MEMS) into the posterior segment via an intraocular cannula inserted through the pars plana. The MEMS enables the precise delivery of small amounts of anti-VEGF agents through the canula. Once depleted, the depot can be refilled via a port. The technology has been tested in a small Phase I clinical trial involving patients with DMO. In the study, the sustained release of Ranibizumab (Lucentis™) was achieved for 90 days without any significant surgical issues, worsening of central foveal thickness on optical coherence tomography or reduction in visual acuity. However, to date, there have not been any larger clinical trials involving the PMP technology [55].

Perhaps the most promising non-biodegradable implant is the port delivery system (PDS), currently being developed by Genentech^©^. It is an implantable, reservoir-based, prolonged-release platform. The PDS is designed to deliver Ranibizumab (Lucentis™) in a concentrated solution at different doses in n-AMD. Surgically implanted to sit in the sclera, the PDS can be refilled with Ranibizumab (Lucentis™) in the clinic. Clinical trials have been promising thus far. In the Phase II long acting delivery of Ranibizumab (LADDER) trial by Genentech^©^, four arms were being evaluated, namely the PDS in 10, 40, 100 mg/ml formulations and monthly 0.5 mg injections. A total of 232 Ranibizumab (Lucentis™) n-AMD patients were recruited for the study. The results demonstrated that 100 mg/ml PDS could produce similar visual acuity outcomes as monthly Ranibizumab (Lucentis™) injections. In the Phase II LADDER trial, the PDS had a median refill time of 15 months [56]. These results have led to the launch of Phase III ARCHWAY trial (ClinicalTrial.gov ID: NCT03677934), a multi-centre, randomized, open-label, study comparing the safety, efficacy and pharmacokinetics of 100 mg/ml PDS with 0.5 mg solution of intravitreal injections. The estimated completion date of the Phase III ARCHWAY trial (ClinicalTrials.gov NCT03677934) should be April 2022. Participants in the Phase III PORTAL extension trial will receive refills every 24 weeks for up to 144 weeks. The estimated completion date of the Phase III PORTAL trial (ClinicalTrials.gov NCT03683251) should be January 2022.

However, the use of PDS exposes the patient to the risk of complications during implantation. While effective at allowing sustained delivery, the device requires an initial procedure involving a scleral wound creation. This is done by performing a stab incision at the pars plana followed by implantation of the device and suturing of the conjunctiva and Tenon’s capsule. In the Phase II LADDER trial, patients with PDS were associated with more adverse events as compared with monthly intravitreal Ranibizumab (Lucentis™) injections due to the surgical nature of the device [56]. Of the 179 PDS-treated patients, 16 (8.9%) had developed ocular serious adverse events, with vitreous haemorrhage being the most common serious adverse event. Although improvements are being made to the PDS, with safety being further assessed in the Phase III ARCHWAY trial, these results provide a reminder of the possible sight-threatening complications that can still arise with a surgical-based sustained drug delivery platform. As such, there is still a role for the development of sustained delivery platforms that do not require major surgical procedures for implantation.

## Nano-formulations for sustained delivery of anti-VEGF

The use of nanotechnology as sustained drug delivery platforms in medicine has been an active research field over the past decade. Nano-formulations are also currently being explored for macromolecule drug delivery to the posterior segment of the eye. There are many various nano-formulations such as polymeric nanoparticles (NPs), liposomes, dendrimers, polymeric micelles, inorganic and carbon nanotubes. Of which, the first three types have been widely investigated as carriers for proteins such as anti-VEGF agents. These nano-formulations share a similar feature; they are usually made of an outer shell that is capable of encapsulating the drug of interest. However, each nano-formulation varies in terms of its structure [57], resulting in slight differences between their characteristics, as explained in greater detail below.

### NPs and microparticles (MPs)

Polymeric NPs and MPs are made of biocompatible and biodegradable polymers, which encapsulate the drug of interest. NPs are typically 1–1000 nm, while MPs are typically 1–1000 µm in size. The outer layer of these NPs and MPs, known as the interfacial layer, is extremely important as it influences the particle’s characteristics. Depending on the polymer that the particle is made of, the interfacial layer can provide the NP/MP delivery system with advantages such as increased tissue penetration, protection of bioactive proteins from enzymatic degradation and extended release of the encapsulated drug.

Polymeric NP and MPs can be made of many materials including natural ones like chitosan or synthetic polymers [58], of which poly(lactide-co-glycolide) (PLGA) is the most widely used biodegradable polymer. PLGA’s biocompatibility and non-toxic degradation products have made it a popular choice for biomedical applications in the human body. To date, many PLGA-based drug delivery systems have been approved by the US-FDA. A PLGA-based intravitreal implant for dexamethasone, Ozurdex™, has already been approved for ocular usage [59].

There has been considerable amount of work done for NP and MP-based anti-VEGF delivery systems in the treatment of neovascularisation of the retina. These systems have been extensively researched as they are capable of encapsulating bioactive drugs, allowing greater drug stability in the targeted tissue. Furthermore, the surface groups on the interfacial layer can be modified to allow functions such as the targeted delivery of NPs, an approach that has been utilised to create targeted therapies in the field of oncology. Despite their advantages, there are currently no NP and MP-based systems that have fulfilled the complete criteria of an ideal sustained anti-VEGF delivery system to the retina. A search of the major clinical trial registries, Clinicaltrials.gov, European Union Clinical Trials Register and the International Standard Randomised Controlled Trial Number Registry, did not yield any clinical trials in this area of research.

Many of the studies have demonstrated that sustained release for anti-VEGF molecules can be achieved for more than 1 month in in vitro and in vivo models [58, 60–64]. However, data regarding the bioactivity exerted by the anti-VEGF loaded particles in reducing retinal neovascularisation in vivo remains limited [58]. In vivo results have mainly been proven in small animals such as rats [58]. In addition, they are hampered by the self-healing observed in many in vivo models such as laser-induced CNV [65], making long-term effects difficult to assess. The majority of the studies have utilised in vitro methods, such as the chick chorioallantoic membrane assay, as surrogates to prove the bioactivity of delivered anti-VEGF [60]. While these experiments can suggest the retention of anti-VEGF bioactivity, appropriate in vivo data using disease specific models is required to demonstrate the ability of NP/MP encapsulated anti-VEGF to regress neovascularisation.

The particle size of NPs and MPs affects the drug loading, the kinetics of sustained release, tissue penetration and biocompatibility. One of the challenges associated with PLGA-based NP/MP systems is creating a particle with an ideal size that balances the above-mentioned characteristics. Particle size controls the rate at which the encapsulated drug is released [66]. In general, with smaller particles, less drug can be loaded. Due to a greater proportion of drug being exposed to the external medium, a small particle size is also associated with both a greater initial burst and subsequent release. As such, small particles often have sustained release durations that are not clinically relevant. On the other hand, small particles offer a higher tissue penetration. This allows the encapsulated anti-VEGF agents to reach the retinal layers. In a study by Sakurai et al., fluorescein-loaded polystyrene nanospheres and microspheres were injected into the vitreous cavity of pigmented rabbit eyes. After a month, MPs were found solely in the vitreous cavity and trabecular meshwork while NPs could also be found in the retina, suggesting a higher penetration as compared with its MP counterpart [67]. With higher penetration, it is also more difficult to predict the eventual fate of small-sized particles. A particular concern is whether these particles will cause unwanted effects in surrounding tissues or even systemically. Unlike other PLGA-based biomedical applications, which have been proven to be safe, the small size of the NP/MP systems have so far limited evidence for biocompatibility and safety [67].

### Liposomes

Another nano-formulation known as the liposome is also increasingly being studied for sustained delivery of anti-VEGF compounds to the posterior segment. Liposomes are made up of a lipid bilayer formed from phospholipids and cholesterol. This bilayer surrounds an aqueous compartment that contains the drug of interest. Both hydrophobic and hydrophilic compounds such as anti-VEGF molecules can be incorporated into the aqueous compartment. As compared with polymeric NP/MP-based delivery systems, liposomes have lower immunogenicity and toxicity as the phospholipid components are readily metabolised once the liposome has disintegrated. Due to its good biocompatibility and ability to encapsulate anti-VEGF drugs, liposomes have also been explored as sustained release delivery platforms.

Various liposome-based systems, when administered intravitreally, have been proven to allow sustained release of anti-VEGF for more than 1 month in animal models with good biocompatibility [65, 68]. Mu et al. have devised a multi-vesicular liposome comprised of a 95:5 ratio of aqueous component to lipid component, allowing a high encapsulation efficiency [65]. This allows the liposome platform to act as a depot for prolonged released of anti-VEGF. The bioactivity of the encapsulated drug, Bevacizumab (Avastin™), was also proven in a Brown-Norway rat model of laser-induced CNV.

Like NP and MP-based systems, the surface of liposomes can be modified to improve characteristics such as tissue penetration. A recent study modified the surface of a phosphatidylserine-based liposome using annexin-A5, to enhance topical delivery of Bevacizumab (Avastin™) across the corneal epithelial barriers to the posterior segment [69]. As compared with the free drug administered topically, the annexin-A5 liposome increased posterior segment delivery by up to threefold. While the actual amount of drug that reached the posterior segment was less than 1% of the clinically administered dose, the results suggest the added potential of liposomes being developed as topical treatments for posterior segment diseases. If proven successful, it may overcome the need for repeated intravitreal injections of anti-VEGF agents.

Despite the above-mentioned advantages, there is still a lack of basic understanding of how liposomes interact with physiological processes. Drug release from liposomes in vivo is a complex phenomenon that is easily affected by various environmental conditions. For instance, liposomes can be destabilised and ingested by macrophages [70]. Furthermore, enzymatic reactions and pH changes can also interfere with liposomal integrity [71]. As there are many factors affecting the stability of the liposome structure in the eye, it is difficult to make general predictions on the duration of sustained delivery. Furthermore, as liposomes exist in suspensions due to their phospholipid content, it has been suggested that their presence in the vitreous can lead to blurring of vision [72].

With these limitations, investigations are still ongoing to optimise the technology for sustained delivery of anti-VEGF molecules to the retina.

### Dendrimers

Dendrimers are polymers composed of repeating, regularly branched units. The dendrimer structure has three major portions: a core, inner branches and outer shell with functional surface groups. As compared with linear polymers, dendrimers have multivalent surface branches. Through functionalization of these surface branches, the properties of the dendrimer can be adjusted accordingly. Some of these properties include tissue targeting and sustained release. Apart from the encapsulation of the drug within the core of the particle, the unique dendrimer structure allows drugs to be loaded via other methods such as direct conjugation and ionic interaction with the surface branches.

While many chemically distinct dendrimers have been synthesized, the field of study is still in its infancy. Few dendrimers have been studied in depth. The polyaminoamine (PAMAM) dendrimer is the most well-studied and well-characterized class of dendrimers to date, due to its biocompatibility and water solubility [73]. Initially explored in the field of targeted therapy in oncology, there is increasing interest in utilising these dendrimers in ocular conditions [73]. A study by Marano et al. demonstrated the application of a lipid–lysine dendrimer conjugated with an anti-VEGF oligonucleotide (ODN-1) in a laser-induced CNV rat model. The lipid–lysine dendrimer was able to encapsulate the oligonucleotide and deliver it through the cell membrane and nucleic membrane of retinal cells to the target site on the DNA strand. In the study, the ODN-1 dendrimer compound lasted up to 6 months in the posterior segment of the eye. This suggests the potential of utilising the ODN-1 dendrimer system as a method for prolonged control of the VEGF pathway in retinal vascular diseases [74]. However, it should be noted that to date a gene therapy approach based on delivery of oligonucleotides to treat retinal vascular diseases has not been approved. Thus, safety of the dendrimer and the oligonucleotide therapeutic would need to be assessed.

The unknown long-term safety profile is the greatest disadvantage of dendrimer systems. In most formulations, dendrimers are small enough to eventually penetrate and enter the systemic circulation. Positively charged dendrimers, which have abundant primary amine end groups, were found to associate strongly with anionic glycans. Glycans are found in the vitreous humour, which translates to a longer sustained duration in the posterior segment. However, they are also present in cellular membranes and dendrimers have been shown to result in cellular membrane disruption [75]. Furthermore, for the purpose of sustained release, biomolecules are generally covalently bonded on the surface of dendrimers via chemical conjugation methods. This may limit the use of dendrimers for anti-VEGF delivery as these chemicals can potentially deactivate the protein. Hence, the technology still requires a significant amount of work before clinical translation can be attained.

#### Hydrogels

A hydrogel is a three-dimensional network of hydrophilic polymers that can swell in water and hold a large amount of water while maintaining its state as a solid [76]. Usually hydrophilic polymers interact with water molecules, resulting in high water solubility. However, in a hydrogel, the hydrophilic polymer chains form a network that retains the favourable water interactions but resist dissolution due to the cross-links between the individual polymer chains. First reported in 1960 by Wichterle and Lim, due to their significant water content, hydrogels possess a degree of flexibility very similar to natural tissue [77]. Recent advances in polymer science have also allowed the creation of “smart” hydrogels that can undergo sol–gel phase transition, swell or degrade in response to various physical and chemical stimuli. This is of great interest to the scientific community due to its potential to be engineered for multiple biomedical applications [78].

Hydrogels are purported to be a promising class of materials for sustained anti-VEGF delivery due to the following reasons. Firstly, the hydrogel’s porous structure provides a matrix for drug loading. This matrix also acts as protection against environmental factors such as enzymatic reactions and hydrophobic interactions, which can cause denaturation of bioactive proteins. This is ideal for anti-VEGF agents, as it protects the quaternary structure, which is crucial for bioactivity. The matrix structure of the hydrogel also allows the loading of a larger amount of drug as compared with nano-formulations. Hence, sustained delivery durations achieved by hydrogel-based systems are significantly longer than nano-formulation systems (Table 2). Furthermore, “smart” hydrogels, which are engineered to sense external environmental triggers such as temperature or pH, also allow an additional level of control over the release of the anti-VEGF molecule [79, 80]. Lastly, due to the large water content, hydrogels are generally highly biocompatible and transparent [81]. These features lend hydrogels great potential as sustained drug delivery systems for anti-VEGF agents in ocular applications [82].

**Fig. 4:**
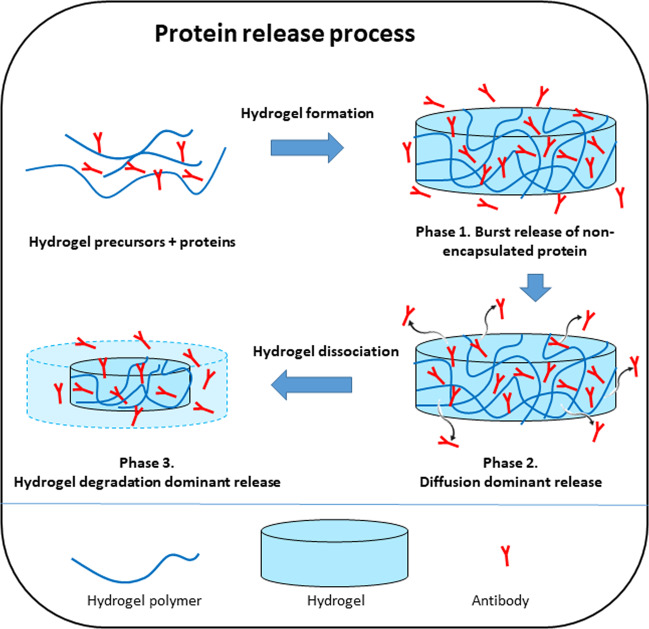
Phases of drug release from hydrogel-based sustained delivery systems. Drug release from hydrogel-based sustained delivery systems can be separated into three phases: an initial burst release phase, diffusion-dominated phase and hydrogel degradation dominated release phase.

The release of bioactive proteins, such as anti-VEGF agents, from the hydrogel can be summarized into three phases: an initial burst release phase, followed by a diffusion-dominated release phase and finally a hydrogel degradation dominated release phase (Fig. 4) [83]. In the burst release phase, before cross-linking of the hydrogel is complete, almost 10–50% of the total protein payload can be lost due to the rapid diffusion of the protein into the vitreous [84, 85]. This is followed by the diffusion-dominated phase, where the protein molecules overcome a series of physical barriers posed by the network structure of polymers in the hydrogel [86–88]. This diffusion is affected by both the concentration of the protein remaining and the pore size of the hydrogel [89]. As the concentration of protein in the gel decreases, the diffusion rate also decreases. Eventually the release is mediated by the degradation of the hydrogel matrix via hydrolysis or enzymatic pathways [90]. Due to the large water content, hydrolysis can occur both at the surface and within the hydrogel, subjecting it to both bulk and surface erosions. When the hydrogel matrix collapses at the final stage, a final burst release of the remaining anti-VEGF occurs.

## Classification of hydrogels based on types of polymer and cross-links

**Fig. 5:**
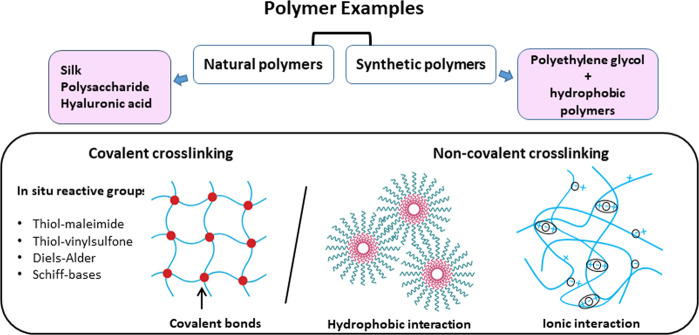
Classification of hydrogels. Hydrogels can be classified either according to the types of polymers that they are made of or the types of cross-links within the gel.

While there are many different ways of classifying hydrogels, a basic classification can be made based on the polymer composition and the types of cross-links (Fig. 5).

## Classifying hydrogels based on polymer type

In general, polymers that are used to make hydrogels for biomedical applications can be classified into three groups: natural polymers, synthetic polymers and hybrid polymers. Natural polymers that have been used for hydrogel production include cellulose, chitosan, alginate, hyaluronic acid and even silk [91, 92]. These polymers are inherently biodegradable due to the presence of hydrolysable linkages within the polymers. The biodegradation process can also be carried out by the enzymes within the human body, and the rate of biodegradation is largely pre-determined and cannot be adjusted.

Natural polymers have been gradually replaced by synthetic polymers over the past 2 decades as the properties of synthetic polymers are tunable by varying the chemical composition and preparation methods. These properties include porosity, swelling ability, stability, mechanical strength and biocompatibility. While there are many biodegradable polymers that have been approved by the US-FDA, the most popular synthetic polymer for hydrogel is polyethylene glycol (PEG), due to its tunable properties and well-established safety profile.

While first discovered in the 1950s, the concept of drug delivery using PEG was only introduced in the 1970s by Abuchowski and Davis [93], and realized in 1990 to treat immunodeficiency disease using a PEG conjugated protein (Adagen™) [94]. Today, PEG is more frequently used for hydrogel formation over other synthetic polymers due to its biocompatibility. Tunable biodegradability can also be conferred by adding ester or urethane bonds into the polymer backbone to allow for hydrolysis [95]. PEG-based hydrogels have been used to encapsulate both hydrophilic and hydrophobic drugs, including hormones such as insulin [96] and steroids [97].

## Classifying hydrogels based on types of cross-linking

Polymers can also be characterized according to the type of cross-linking. In general, these cross-links can be divided into non-covalent cross-links and covalent cross-links. The forces involved in the formation of non-covalent cross-links include hydrophobic, electrostatic and hydrogen bonds between polymer chains. As these forces are generally weak, they are easily reversible by environmental alterations such as pH or temperature. Hence, hydrogels with non-covalent cross-links are also known as reversible hydrogels. These hydrogels are prepared without the use of cross-linking entities or chemical modification. On the other hand, covalent cross-linked hydrogels involve the covalent bonding between polymer chains. The formation of these cross-links can be achieved by adding small cross-linking molecules, polymer–polymer conjugation, photosensitive agents or utilizing enzyme catalysed reactions.

For the functional incorporation of bioactive molecules such as anti-VEGF compounds, non-covalent cross-linked and covalent cross-linked hydrogels have their own set of advantages and disadvantages. Covalent cross-linked hydrogels require additional chemical reactions, which often result in the modification and inactivation of bioactive molecules. Addition of cross-linking agents can also result in increased toxicity. On the other hand, the variables required for a successful sustained drug release platform such as gelation time and diffusion rate are difficult to fine tune in non-covalent cross-linked hydrogels as their effects are generally difficult to predict.

## Hydrogels for sustained delivery of anti-VEGF

Hydrogels are ideal sustained delivery platforms for anti-VEGFs because they are capable of packaging a high payload of bioactive hydrophilic drugs, sustaining anti-VEGF release for multiple months. Furthermore, they are transparent, biocompatible and able to gelate without using cross-linking agents, which may denature the bioactive proteins [91, 92]. Various hydrogel variations have been tested for sustained release of anti-VEGF compounds in the eye.

Most hydrogel platforms have not only shown to be biocompatible but also allow a sustained release of bioactive anti-VEGF to the retina for durations of multiple months [91, 92, 98–102] (Table 2). Yu et al. developed a hydrogel system comprised of natural polymers. The vinylsulfone functionalized hyaluronic acid and thiolated dextran (Dex-SH) hydrogel was capable of sustaining the release of Bevacizumab (Avastin™) in rabbit eyes for more than 6 months. This hydrogel also demonstrated good biocompatibility and transparency in situ when indirect ophthalmoscopy was performed [92]. Another group, Lovett et al., reported a silk-based hydrogel that demonstrated a sustained release of Bevacizumab (Avastin™) for at least 3 months in rabbit models with good biocompatibility [91]. Importantly, the hydrogels were capable of sustaining therapeutically relevant concentrations (~50 μg/ml) of Bevacizumab (Avastin™) and the volume of gel injected was less than the volume of Bevacizumab (Avastin™) injected in the clinical setting.

As an added advantage, apart from being utilized as a sustained drug release platform, the hydrogel’s gel state can be concurrently leveraged for other purposes in the posterior chamber. Thermo-responsive gels can also be utilized as vitreo-tamponade agents in the treatment of retinal detachment. In a study by Liu et al., a urethane-based thermogelling polymer was able to achieve retinal tamponade in non-human primate vitrectomy models for up to 12 months [47]. Future applications include the incorporation of anti-VEGFs into these gels to serve the dual function of vitreous tamponade and drug delivery. This will be particularly useful for patients with PDR requiring vitrectomy surgery.

When hydrogel-based delivery systems were first explored for ocular drug delivery, the loss of loaded protein or drug during the initial burst release phase was a significant challenge. For instance, in a chemically cross-linked PEG hydrogel containing Bevacizumab (Avastin™) developed by Yu et al., 25% of the loaded drug was observed to be released on day 1 during the initial burst release phase, resulting in a sustained delivery in vitro for only up to 14 days [85]. In an attempt to prolong the drug release period, other groups have modified hydrogel polymers to be more resistant to degradation. Kichhoff et al. have demonstrated this by creating an eight-arm PEG hydrogel and adding a hydrophobic 6-aminohexanoic acid spacer between the PEG and reactive end groups for increased stability. This resulted in reduced burst release, and prolonged degradation time, with sustained release of anti-VEGF for up to 6 weeks in vitro [88]. The hydrogel is currently being validated for biocompatibility in in vivo models of choroidal neovascularization. Another strategy attempted was to embed a NP/MP-based delivery system into a hydrogel system [103, 104]. By embedding anti-VEGF filled PLGA microspheres in a thermo-responsive PNIPAAm-PEG-diacrylate hydrogel, Osswald et al. were able to achieve a sustained release of Ranibizumab (Lucentis™) and Aflibercept (Eylea™) for almost 200 days in vitro [105]. Bioactivity of the anti-VEGF agents was also retained as evidenced by the significantly smaller CNV lesion areas in the treated CNV rat models of the study [99].

Today, hydrogel platforms face a more downstream challenge in the clinical translation process. As it is a relatively novel technology, the manufacturing process possesses significant barriers to translation. Sterilization is one of these major barriers. As part of the clinical translation process, sterility is required to ensure the product does not introduce any infection into the targeted tissue. Common sterilization methods include using heat, radiation or chemicals. However, these methods are often too harsh. Choosing an appropriate sterilization method is important as exposure to harsh methods can alter the function of the hydrogel and its payload. Hydrogels, in particular, are susceptible to degradation, decomposition and further cross-linking. This changes the hydrogel’s physical properties, which have been proven to work in vitro and in vivo. To overcome this, Ji and Shi have attempted to combine the fabrication and sterilization process. Using steam sterilization, sterile aspirin-loaded chitosan hydrogels were fabricated without an extra sterilization step, making them ready for direct clinical trial usage. This process was also organic solvent-free, reducing the risk of payload modification and residual toxic solvents [106]. To date, several labs have managed to produce various sterilized hydrogel formulations [106, 107].

The progress of hydrogels in this field may be more developed than described in academic reports. Even the above-mentioned manufacturing challenges may have already been tackled by solutions in the industry. Ocular Therapeutix^TM^, a biopharmaceutical company that holds a proprietary hydrogel-based technology, has recently started an anti-VEGF sustained delivery program in collaboration with Regeneron^TM^. Labelled as OTX-IVT, the hydrogel is an injectable, biodegradable shape-changing hydrogel implant loaded with pharmaceuticals. After injection, the hydrogel resides free floating within the vitreous and eventually dissolves after 6–7 months. While details of the technology have been scant due to proprietary issues, the company has showcased the ability of OTX-IVT to enable sustained release of bioactive anti-VEGF to the posterior segment of the eye [108]. When loaded with Bevacizumab (Avastin™), the OTX-IVT was able to inhibit vascular leakage in a rabbit VEGF challenge model for up to 12 weeks as evidenced on fluorescein angiography. The hydrogel also had good biocompatibility. While the anti-VEGF technology is currently still a pre-clinical program, the same technology has been utilized in a Phase I clinical trial for the delivery of tyrosine kinase inhibitors in patients with n-AMD (ClinicalTrial.gov ID: NCT03630315). The interest from the industry in developing hydrogels for this purpose highlights the proximity of hydrogels to achieving clinical utility as compared with nano-formulations.

## Conclusion and future perspectives

There remains an unmet clinical need for sustained anti-VEGF delivery systems for the posterior segment of the eye. The development of such drug delivery systems remains challenging due to the need to encapsulate sufficient anti-VEGF in a small volume (<0.1 ml), whilst ensuring sustained release of anti-VEGF with preserved bioactivity in vivo. Of the various innovative research strategies, non-biodegradable implants have proven themselves to be the closest to reaching clinical approval. However, they are associated with complications from the surgical implant procedure. Hydrogels are by far the most promising candidate with recent publications indicating good biocompatibility and safety, with upcoming industry driven clinical trials.

## References

[1] Michels S, Rosenfeld PJ, Puliafito CA, Marcus EN, Venkatraman AS (2005). Systemic bevacizumab (Avastin) therapy for neovascular age-related macular degeneration twelve-week results of an uncontrolled open-label clinical study. Ophthalmology..

[2] Lau PE, Jenkins KS, Layton CJ. Current evidence for the prevention of endophthalmitis in anti-VEGF intravitreal injections. J Ophthalmol. 2018;2018:8567912. 10.1155/2018/8567912 PMC6098904 30174946

[3] Mason JO, White MF, Feist RM, Thomley ML, Albert MA, Persaud TO (2008). Incidence of acute onset endophthalmitis following intravitreal bevacizumab (Avastin) injection. Retina.

[4] Bhavsar AR, Googe JM, Stockdale CR, Bressler NM, Brucker AJ, Elman MJ (2009). Risk of endophthalmitis after intravitreal drug injection when topical antibiotics are not required: the diabetic retinopathy clinical research network laser-ranibizumab-triamcinolone clinical trials. Arch Ophthalmol..

[5] Spooner KL, Mhlanga CT, Hong TH, Broadhead GK, Chang AA (2018). The burden of neovascular age-related macular degeneration: a patient’s perspective. Clin Ophthalmol.

[6] Prenner JL, Halperin LS, Rycroft C, Hogue S, Williams Liu Z, Seibert R (2015). Disease burden in the treatment of age-related macular degeneration: findings from a Time-and-Motion Study. Am J Ophthalmol..

[7] Wong WL, Su X, Li X, Cheung CM, Klein R, Cheng CY (2014). Global prevalence of age-related macular degeneration and disease burden projection for 2020 and 2040: a systematic review and meta-analysis. Lancet Glob Health.

[8] Lee R, Wong TY, Sabanayagam C (2015). Epidemiology of diabetic retinopathy, diabetic macular edema and related vision loss. Eye Vis.

[9] Huang H, He J, Johnson D, Wei Y, Liu Y, Wang S (2015). Deletion of placental growth factor prevents diabetic retinopathy and is associated with Akt activation and HIF1alpha-VEGF pathway inhibition. Diabetes..

[10] Wisniewska-Kruk J, Hoeben KA, Vogels IM, Gaillard PJ, Van Noorden CJ, Schlingemann RO (2012). A novel co-culture model of the blood-retinal barrier based on primary retinal endothelial cells, pericytes and astrocytes. Exp Eye Res.

[11] Wells JA, Glassman AR, Ayala AR, Jampol LM, Bressler NM, Bressler SB (2016). Aflibercept, bevacizumab, or ranibizumab for diabetic macular edema: two-year results from a comparative effectiveness randomized clinical trial. Ophthalmology..

[12] Campochiaro PA, Heier JS, Feiner L, Gray S, Saroj N, Rundle AC (2010). Ranibizumab for macular edema following branch retinal vein occlusion: six-month primary end point results of a phase III study. Ophthalmology..

[13] Campochiaro PA, Clark WL, Boyer DS, Heier JS, Brown DM, Vitti R (2015). Intravitreal aflibercept for macular edema following branch retinal vein occlusion: the 24-week results of the VIBRANT study. Ophthalmology..

[14] Brown DM, Campochiaro PA, Singh RP, Li Z, Gray S, Saroj N (2010). Ranibizumab for macular edema following central retinal vein occlusion: six-month primary end point results of a phase III study. Ophthalmology..

[15] Heier JS, Clark WL, Boyer DS, Brown DM, Vitti R, Berliner AJ (2014). Intravitreal aflibercept injection for macular edema due to central retinal vein occlusion: two-year results from the COPERNICUS study. Ophthalmology..

[16] Ogura Y, Roider J, Korobelnik JF, Holz FG, Simader C, Schmidt-Erfurth U (2014). Intravitreal aflibercept for macular edema secondary to central retinal vein occlusion: 18-month results of the phase 3 GALILEO study. Am J Ophthalmol.

[17] Noma H, Funatsu H, Mimura T, Eguchi S, Shimada K (2011). Role of soluble vascular endothelial growth factor receptor-2 in macular oedema with central retinal vein occlusion. Br J Ophthalmol.

[18] Kwak N, Okamoto N, Wood JM, Campochiaro PA (2000). VEGF is major stimulator in model of choroidal neovascularization. Investig Ophthalmol Vis Sci.

[19] Chakravarthy U, Harding SP, Rogers CA, Downes SM, Lotery AJ, Wordsworth S (2012). Ranibizumab versus bevacizumab to treat neovascular age-related macular degeneration: one-year findings from the IVAN randomized trial. Ophthalmology..

[20] Heier JS, Brown DM, Chong V, Korobelnik JF, Kaiser PK, Nguyen QD (2012). Intravitreal aflibercept (VEGF trap-eye) in wet age-related macular degeneration. Ophthalmology..

[21] Gragoudas ES, Adamis AP, Cunningham ET, Feinsod M, Guyer DR (2004). Pegaptanib for neovascular age-related macular degeneration. N Engl J Med.

[22] Rosenfeld PJ, Moshfeghi AA, Puliafito CA (2005). Optical coherence tomography findings after an intravitreal injection of bevacizumab (avastin) for neovascular age-related macular degeneration. Ophthalmic Surg, Lasers Imaging.

[23] Falavarjani KG, Nguyen QD (2013). Adverse events and complications associated with intravitreal injection of anti-VEGF agents: a review of literature. Eye.

[24] Edward A, Prausnitz MR (2001). Predicted permeability of the cornea to topical drugs. Pharm Res..

[25] Ahmed I, Patton TF (1985). Importance of the noncorneal absorption route in topical ophthalmic drug delivery. Investig Ophthalmol Vis Sci.

[26] Lee J, Pelis RM (2016). Drug transport by the blood-aqueous humor barrier of the eye. Drug Metab Dispos.

[27] Ambati J, Canakis CS, Miller JW, Gragoudas ES, Edwards A, Weissgold DJ (2000). Diffusion of high molecular weight compounds through sclera. Investig Ophthalmol Vis Sci.

[28] Edington M, Connolly J, Chong NV (2017). Pharmacokinetics of intravitreal anti-VEGF drugs in vitrectomized versus non-vitrectomized eyes. Expert Opin Drug Metab Toxicol.

[29] Patel S (2018). Medicare spending on anti-vascular endothelial growth factor medications. Ophthalmol Retin.

[30] Holz FG, Tadayoni R, Beatty S, Berger A, Cereda MG, Cortez R (2015). Multi-country real-life experience of anti-vascular endothelial growth factor therapy for wet age-related macular degeneration. Br J Ophthalmol.

[31] Ehlken C, Helms M, Bohringer D, Agostini HT, Stahl A (2018). Association of treatment adherence with real-life VA outcomes in AMD, DME, and BRVO patients. Clin Ophthalmol.

[32] Kiss S, Liu Y, Brown J, Holekamp NM, Almony A, Campbell J (2014). Clinical utilization of anti-vascular endothelial growth-factor agents and patient monitoring in retinal vein occlusion and diabetic macular edema. Clin Ophthalmol.

[33] Jumper JM, Dugel PU, Chen S, Blinder KJ, Walt JG (2018). Anti-VEGF treatment of macular edema associated with retinal vein occlusion: patterns of use and effectiveness in clinical practice (ECHO study report 2). Clin Ophthalmol.

[34] Holekamp NM, Liu Y, Yeh WS, Chia Y, Kiss S, Almony A (2014). Clinical utilization of anti-VEGF agents and disease monitoring in neovascular age-related macular degeneration. Am J Ophthalmol.

[35] Holekamp NM, Campbell J, Almony A, Ingraham H, Marks S, Chandwani H (2018). Vision outcomes following anti-vascular endothelial growth factor treatment of diabetic macular edema in clinical practice. Am J Ophthalmol.

[36] Blinder KJ, Dugel PU, Chen S, Jumper JM, Walt JG, Hollander DA (2017). Anti-VEGF treatment of diabetic macular edema in clinical practice: effectiveness and patterns of use (ECHO Study Report 1). Clin Ophthalmol.

[37] Brown DM, Kaiser PK, Michels M, Soubrane G, Heier JS, Kim RY (2006). Ranibizumab versus verteporfin for neovascular age-related macular degeneration. N. Engl J Med.

[38] Kaiser PK, Boyer DS, Cruess AF, Slakter JS, Pilz S, Weisberger A (2012). Verteporfin plus ranibizumab for choroidal neovascularization in age-related macular degeneration: twelve-month results of the DENALI study. Ophthalmology..

[39] Larsen M, Schmidt-Erfurth U, Lanzetta P, Wolf S, Simader C, Tokaji E (2012). Verteporfin plus ranibizumab for choroidal neovascularization in age-related macular degeneration: twelve-month MONT BLANC study results. Ophthalmology..

[40] Dugel PU, Bebchuk JD, Nau J, Reichel E, Singer M, Barak A (2013). Epimacular brachytherapy for neovascular age-related macular degeneration: a randomized, controlled trial (CABERNET). Ophthalmology..

[41] Arnold JJ, Campain A, Barthelmes D, Simpson JM, Guymer RH, Hunyor AP (2015). Two-year outcomes of “treat and extend” intravitreal therapy for neovascular age-related macular degeneration. Ophthalmology..

[42] Ohnaka MOM, Okada AA, Terano Y, Kobayashi M, Takahashi K. Randomised, open-label study to evaluate 2 intravitreal aflibercept treat-and-extend dosing regimens in wet age-related macular degeneration: 52-week outcomes from altair. In 49th Annual Scientific Congress, The Royal Australian and New Zealand College of Ophthalmolgists. Clinical and Experimental Ophthalmology. 2017;45(Suppl 1):18–33.

[43] Chang A, Warburton J, Weichselberger A, Sallstigo P. Phase iii studies comparing the efficacy and safety of brolucizumab vs aflibercept in subjects with neovascular age-related macular degeneration: testing an alternative treatment regimen. Clinical and Experimental Ophthalmology. 2016;44(Suppl 1):80–140.

[44] Dugel PU, Koh A, Ogura Y, Jaffe GJ, Schmidt-Erfurth U, Brown DM, et al. HAWK and HARRIER: phase 3, multicenter, randomized, double-masked trials of brolucizumab for neovascular age-related macular degeneration. Ophthalmology. 2020;127:72–84. 10.1016/j.ophtha.2019.04.017 30986442

[45] Xue K, Zhao X, Zhang Z, Qiu B, Tan QSW, Ong KH, et al. Sustained delivery of anti-VEGFs from thermogel depots inhibits angiogenesis without the need for multiple injections. Biomater Sci. 2019;7:4603–14. 10.1039/c9bm01049a 31436780

[46] Xue K, Wang X, Yong PW, Young DJ, Wu YL, Li Z (2019). Hydrogels as emerging materials for translational biomedicine. Adv Ther.

[47] Liu Z, Liow SS, Lai SL, Alli-Shaik A, Holder GE, Parikh BH, et al. Retinal-detachment repair and vitreous-like-body reformation via a thermogelling polymer endotamponade. Nat Biomed Eng. 2019;3:598–610. 10.1038/s41551-019-0382-7 30962587

[48] Zhang ZX, Young DJ, Li Z, Loh XJ (2019). Going beyond traditional applications? The potential of hydrogels. small. Methods..

[49] Chan SY, Chan BQY, Liu Z, Parikh BH, Zhang K, Lin Q (2017). Electrospun pectin-polyhydroxybutyrate nanofibers for retinal tissue engineering. ACS Omega..

[50] Liu Z, Su X, Tan MJ, Li Z, Lakshminarayanan R, Barathi VA (2016). Engineering an injectable thermosensitive hydrogel as an internal tamponading agent for vitreo-retinal surgery. Investig Ophthalmol Vis Sci..

[51] Su X, Tan MJ, Li Z, Wong M, Rajamani L, Lingam G (2015). Recent progress in using biomaterials as vitreous substitutes. Biomacromolecules..

[52] Xue K, Liow SS, Karim AA, Li Z, Loh XJ (2018). A recent perspective on noncovalently formed polymeric hydrogels. Chem Rec.

[53] Lakshminarayanan R, Barathi VA, Venkatesh M, Verma NK, Liu S, Loh XJ (2016). Membrane selectivity of cationic polyamides and rational design of proteolytic-resistant antimicrobial peptides. Investig Ophthalmol Vis Sci..

[54] Oo C, Kalbag SS (2016). Leveraging the attributes of biologics and small molecules, and releasing the bottlenecks: a new wave of revolution in drug development. Expert Rev Clin Pharmacol..

[55] Humayun M, Santos A, Altamirano JC, Ribeiro R, Gonzalez R, de la Rosa A (2014). Implantable MicroPump for drug delivery in patients with diabetic macular edema. Transl Vis Sci Technol.

[56] Campochiaro PA, Marcus DM, Awh CC, Regillo C, Adamis AP, Bantseev V, et al. The port delivery system with ranibizumab for neovascular age-related macular degeneration: results from the randomized phase 2 ladder clinical trial. Ophthalmology. 2019;126:1141–54. 10.1016/j.ophtha.2019.03.036 30946888

[57] Kai D, Ren W, Tian L, Chee PL, Liu Y, Ramakrishna S (2016). Engineering poly (lactide)–lignin nanofibers with antioxidant activity for biomedical application. ACS Sustain Chem Eng.

[58] Lu Y, Zhou N, Huang X, Cheng JW, Li FQ, Wei RL (2014). Effect of intravitreal injection of bevacizumab-chitosan nanoparticles on retina of diabetic rats. Int J Ophthalmol.

[59] Boyer DS, Yoon YH, Belfort R, Bandello F, Maturi RK, Augustin AJ (2014). Three-year, randomized, sham-controlled trial of dexamethasone intravitreal implant in patients with diabetic macular edema. Ophthalmology..

[60] Liu J, Li S, Li G, Li X, Yu C, Fu Z (2019). Highly bioactive, bevacizumab-loaded, sustained-release PLGA/PCADK microspheres for intravitreal therapy in ocular diseases. Int J Pharm.

[61] Varshochian R, Riazi-Esfahani M, Jeddi-Tehrani M, Mahmoudi AR, Aghazadeh S, Mahbod M (2015). Albuminated PLGA nanoparticles containing bevacizumab intended for ocular neovascularization treatment. J Biomed Mater Res Part A.

[62] Sun JG, Jiang Q, Zhang XP, Shan K, Liu BH, Zhao C (2019). Mesoporous silica nanoparticles as a delivery system for improving antiangiogenic therapy. Int J Nanomed.

[63] Ye Z, Ji YL, Ma X, Wen JG, Wei W, Huang SM (2015). Pharmacokinetics and distributions of bevacizumab by intravitreal injection of bevacizumab-PLGA microspheres in rabbits. Int J Ophthalmol.

[64] Yandrapu SK, Upadhyay AK, Petrash JM, Kompella UB (2013). Nanoparticles in porous microparticles prepared by supercritical infusion and pressure quench technology for sustained delivery of bevacizumab. Mol Pharm..

[65] Mu H, Wang Y, Chu Y, Jiang Y, Hua H, Chu L (2018). Multivesicular liposomes for sustained release of bevacizumab in treating laser-induced choroidal neovascularization. Drug Deliv..

[66] Robinson R, Viviano SR, Criscione JM, Williams CA, Jun L, Tsai JC (2011). Nanospheres delivering the EGFR TKI AG1478 promote optic nerve regeneration: the role of size for intraocular drug delivery. ACS Nano..

[67] Sakurai E, Ozeki H, Kunou N, Ogura Y (2001). Effect of particle size of polymeric nanospheres on intravitreal kinetics. Ophthalmic Res..

[68] Abrishami M, Zarei-Ghanavati S, Soroush D, Rouhbakhsh M, Jaafari MR, Malaekeh-Nikouei B (2009). Preparation, characterization, and in vivo evaluation of nanoliposomes-encapsulated bevacizumab (avastin) for intravitreal administration. Retina.

[69] Davis BM, Normando EM, Guo L, Turner LA, Nizari S, O’Shea P (2014). Topical delivery of Avastin to the posterior segment of the eye in vivo using annexin A5-associated liposomes. Small.

[70] Moghimi SM, Patel HM (1989). Serum opsonins and phagocytosis of saturated and unsaturated phospholipid liposomes. Biochim Biophys Acta.

[71] Yatvin MB, Kreutz W, Horwitz BA, Shinitzky M (1980). pH-sensitive liposomes: possible clinical implications. Science.

[72] Honda M, Asai T, Oku N, Araki Y, Tanaka M, Ebihara N (2013). Liposomes and nanotechnology in drug development: focus on ocular targets. Int J Nanomed.

[73] Chaplot SP, Rupenthal ID (2014). Dendrimers for gene delivery–a potential approach for ocular therapy?. J Pharm Pharmacol.

[74] Marano RJ, Toth I, Wimmer N, Brankov M, Rakoczy PE (2005). Dendrimer delivery of an anti-VEGF oligonucleotide into the eye: a long-term study into inhibition of laser-induced CNV, distribution, uptake and toxicity. Gene Ther..

[75] Yavuz B, Pehlivan SB, Unlu N (2013). Dendrimeric systems and their applications in ocular drug delivery. Sci World J.

[76] Li J, Mooney DJ (2016). Designing hydrogels for controlled drug delivery. Nat Rev Mater.

[77] Wichterle O, LÍM D (1960). Hydrophilic gels for biological use. Nature..

[78] Chai Q, Jiao Y, Yu X. Hydrogels for biomedical applications: their characteristics and the mechanisms behind them. Gels. 2017;3. 10.3390/gels3010006 PMC6318667 30920503

[79] Kang Derwent JJ, Mieler WF (2008). Thermoresponsive hydrogels as a new ocular drug delivery platform to the posterior segment of the eye. Trans Am Ophthalmol Soc.

[80] Matanovic MR, Kristl J, Grabnar PA (2014). Thermoresponsive polymers: insights into decisive hydrogel characteristics, mechanisms of gelation, and promising biomedical applications. Int J Pharm.

[81] Anwary M, Kumar P, du Toit LC, Choonara YE, Pillay V (2018). Polymeric, injectable, intravitreal hydrogel devices for posterior segment applications and interventions. Artif Cells, Nanomed, Biotechnol.

[82] Lau CML, Yu Y, Jahanmir G, Chau Y (2018). Controlled release technology for anti-angiogenesis treatment of posterior eye diseases: current status and challenges. Adv Drug Deliv Rev.

[83] Lin CC, Metters AT (2006). Hydrogels in controlled release formulations: network design and mathematical modeling. Adv Drug Deliv Rev.

[84] Huang X, Brazel CS (2001). On the importance and mechanisms of burst release in matrix-controlled drug delivery systems. J Control Release.

[85] Yu J, Xu X, Yao F, Luo Z, Jin L, Xie B (2014). In situ covalently cross-linked PEG hydrogel for ocular drug delivery applications. Int J Pharm.

[86] Censi R, Vermonden T, van Steenbergen MJ, Deschout H, Braeckmans K, De Smedt SC (2009). Photopolymerized thermosensitive hydrogels for tailorable diffusion-controlled protein delivery. J Control Release.

[87] Mellott MB, Searcy K, Pishko MV (2001). Release of protein from highly cross-linked hydrogels of poly(ethylene glycol) diacrylate fabricated by UV polymerization. Biomaterials..

[88] Kirchhof S, Gregoritza M, Messmann V, Hammer N, Goepferich AM, Brandl FP (2015). Diels-Alder hydrogels with enhanced stability: first step toward controlled release of bevacizumab. Eur J Pharm Biopharm.

[89] Weber LM, Lopez CG, Anseth KS (2009). Effects of PEG hydrogel crosslinking density on protein diffusion and encapsulated islet survival and function. J Biomed Mater Res Part A.

[90] Ikada Y, Tabata Y (1998). Protein release from gelatin matrices. Adv drug Deliv Rev.

[91] Lovett ML, Wang X, Yucel T, York L, Keirstead M, Haggerty L (2015). Silk hydrogels for sustained ocular delivery of anti-vascular endothelial growth factor (anti-VEGF) therapeutics. Eur J Pharm Biopharm.

[92] Yu Y, Lau LC, Lo AC, Chau Y (2015). Injectable chemically crosslinked hydrogel for the controlled release of bevacizumab in vitreous: a 6-month in vivo study. Transl Vis Sci Technol..

[93] Abuchowski A, McCoy JR, Palczuk NC, van Es T, Davis FF (1977). Effect of covalent attachment of polyethylene glycol on immunogenicity and circulating life of bovine liver catalase. J Biol Chem.

[94] Booth C, Gaspar HB (2009). Pegademase bovine (PEG-ADA) for the treatment of infants and children with severe combined immunodeficiency (SCID). Biologics.

[95] Yu L, Ding J (2008). Injectable hydrogels as unique biomedical materials. Chem Soc Rev.

[96] Rong X, Ji Y, Zhu X, Yang J, Qian D, Mo X (2019). Neuroprotective effect of insulin-loaded chitosan nanoparticles/PLGA-PEG-PLGA hydrogel on diabetic retinopathy in rats. Int J Nanomed.

[97] Lu C, Zahedi P, Forman A, Allen C (2014). Multi-arm PEG/silica hydrogel for sustained ocular drug delivery. J Pharm Sci.

[98] Rauck BM, Friberg TR, Medina Mendez CA, Park D, Shah V, Bilonick RA (2014). Biocompatible reverse thermal gel sustains the release of intravitreal bevacizumab in vivo. Investig Ophthalmol Vis Sci.

[99] Osswald CR, Guthrie MJ, Avila A, Valio JA, Mieler WF, Kang-Mieler JJ (2017). In vivo efficacy of an injectable microsphere-hydrogel ocular drug delivery system. Curr Eye Res.

[100] Xie B, Jin L, Luo Z, Yu J, Shi S, Zhang Z (2015). An injectable thermosensitive polymeric hydrogel for sustained release of Avastin(R) to treat posterior segment disease. Int J Pharm.

[101] Park D, Shah V, Rauck BM, Friberg TR, Wang Y (2013). An anti-angiogenic reverse thermal gel as a drug-delivery system for age-related wet macular degeneration. Macromol Biosci..

[102] Xue K, Zhao X, Zhang Z, Qiu B, Tan QSW, Ong KH (2019). Sustained delivery of anti-VEGFs from thermogel depots inhibits angiogenesis without the need for multiple injections. Biomater Sci.

[103] Liu W, Lee BS, Mieler WF, Kang-Mieler JJ (2019). Biodegradable microsphere-hydrogel ocular drug delivery system for controlled and extended release of bioactive aflibercept in vitro. Curr Eye Res.

[104] Liu W, Borrell MA, Venerus DC, Mieler WF, Kang-Mieler JJ (2019). Characterization of biodegradable microsphere-hydrogel ocular drug delivery system for controlled and extended release of ranibizumab. Transl Vis Sci Technol..

[105] Osswald CR, Kang-Mieler JJ (2016). Controlled and extended in vitro release of bioactive anti-vascular endothelial growth factors from a microsphere-hydrogel drug delivery system. Curr Eye Res.

[106] Ji C, Shi J (2012). Sterilization-free chitosan hydrogels for controlled drug release. Mater Lett..

[107] Ferraz CC, Varca GHC, Lopes PS, Mathor MB, Lugão AB (2014). Radio-synthesized polyacrylamide hydrogels for proteins release. Radiat Phys Chem.

[108] Ocular Therapeutix. Intravitreal depot technology for retinal drug delivery: ophthalmology innovation summits 2019. https://ois.net/ocular-therapeutix-intravitreal-depot-technology-for-retinal-drug-delivery/.

